# Using Digital Technology to Design a Simple Interactive System for Nostalgic Gaming to Promote the Health of Slightly Disabled Elderly People

**DOI:** 10.3390/ijerph20010128

**Published:** 2022-12-22

**Authors:** Chao-Ming Wang, Chen-Siang Huang

**Affiliations:** Department of Digital Media Design, National Yunlin University of Science and Technology, Douliu 64002, Taiwan

**Keywords:** successful aging, interactive technology, digital game, disabled elderly adults, usability evaluations, health promotion

## Abstract

An interactive digital gaming system with simple tangible interfaces is proposed for use by slightly disabled elderly people to promote their health and enjoyment of playful aging. The system simulates a rice threshing machine with nostalgic and entertaining functions expected to bring better life quality to older adults. Initially, pieces of literature were reviewed to derive relevant design principles. A prototype system was constructed accordingly and refined according to the invited older users’ comments. The refined system was performed subsequently by slightly disabled elderly people, followed by a questionnaire survey conducted to collect their opinions. The opinion data were analyzed statistically by SPSS and AMOS to be reliable and valid. In addition, interviews were conducted with the users and experts were invited to collect comments on the system’s usability, which were then evaluated to reveal several findings about the system’s effectiveness: (1) digital products related to life experiences are more acceptable to slightly disabled elderly people, promoting their willingness to play games to achieve active aging; (2) simple system interfaces requiring no complicated limb functions are appropriate for the slightly disabled elderly people; and (3) digital gaming has the effects of training slightly disabled elderly peoples’ cognitive and motor abilities as well as strengthening their body and mind.

## 1. Introduction

A person aged 65 or over is regarded as an older adult, and a country is said to turn into an aged society when the older adults in the population of the country reaches 14% [1]. Accordingly, Taiwan became an aged society in 2018 [2], and many problems have arisen in which the main issue is how to help elderly people to go through a healthy aging process by providing them with better healthcare services.

With the development of technology, digital products are ubiquitous nowadays; it is impossible for older adults to avoid facing them in daily life. It has been pointed out in many studies [3,4] that many older adults cannot adapt to the development of new technology and have difficulties in using new technology devices due to the declination of their capabilities of perception and cognition. Such difficulties in general come from the complexity of the user interfaces of such products as well as the elderly users’ lack of experiences in product use, not just because of the aging effect [5].

Contrarily, providing technology products for older adults to use properly in daily life can improve the quality of their life [6,7], helping them to create the feelings of self-satisfaction and happiness [8,9]. Related studies have revealed that regular exercise can promote older adults’ physical fitness and increase their muscle strength, helping them to enjoy a healthy and active aging life [10,11]. Many related studies have been conducted in this aspect to help older adults to conduct exercises by use of ICT (information and communication) products, including the projects of iStoppFalls, FARSEEING, and PreventIT carried out by several international research groups in Europe [12,13,14].

Digital games can help maintain and improve older adults’ health and life quality [15,16,17,18]. Specifically, by playing games for fun and amusement, an older adult can gain new knowledge, conduct exercise, and become relaxed in life. Some recently commonly seen digital games such as Wiimote [19] and Kinect [20] are played by use of human body movements such as swinging and jumping, which can help older adults achieve the effect of joy and fitness. However, the subjects of this study are *slightly disabled elderly people*, who are capable of conducting *activities of daily living* (ADL) that are oriented toward taking care of one’s own body according to Rogers and Holm [21] and they can move freely with their hands and feet but with relative slow movements and no jumping. Therefore, games played by use of Wiimote and Kinect that require fast limb movements or fine finger operations are *not* appropriate for slightly disabled elderly people.

In addition, helping the slightly disabled elderly people to become interested in exercise and engaged in them regularly for successful aging is worth investigating. For this aim, the approach of providing amusement and recreation to slightly disabled older adults is adopted in this study, which is fulfilled by designing an interactive system that provides a user-friendly *nostalgic game* for use by slightly disabled elderly people. Here, a nostalgic game means a simulation of an activity conducted by people in the old days, which can be performed easily by a player to arouse his/her memories of good times in the earlier life.

More specifically, in this study the following related issues are investigated.

(1) How to design an interactive system suitable for use by slightly disabled elderly people in order to introduce digital technology into their daily lives.
(2) How to design simple system interfaces which can be performed by slightly disabled elderly people smoothly with no pressure.
(3) How to apply digital gaming technology to promote the positive effectiveness of leisure and entertainment for slightly disabled elderly people.

In addition, owing to the importance of rice as one of the main foods consumed by people, the nostalgic situations of rice planting and harvesting were introduced into the interactive gaming system designed in this study for use by the slightly disabled elderly people to promote their health as well as life quality during their aging process.

## 2. Literature Review

In this section, existing investigations about interactive system designs for use by older adults are reviewed, and the proposed interactive system is described briefly.

### 2.1. Using ICT for Successful Aging

Due to aging, older adults often encounter inconveniences or difficulties in using technology products. Zajicek [22] mentioned that older adults tend to forget the complicated procedures involved in using a computer and often worry about damaging the system, so they are reluctant to use high-tech products or are too cautious when using them. To compensate the loss of confidence in life faced by most older adults, the concept of active aging has been proposed by the WHO, emphasizing the idea of lifelong learning as one of the central issues involved in the active aging process [23,24]. In addition, many scholars have advocated the idea of successful aging and similar concepts which are useful for older adults to face the aging process positively and aggressively [25,26,27,28,29,30,31,32,33,34,35]. It is beneficial to slightly disabled elderly adults to design aging-related ICT products for use by them to meet their healthcare needs and slow down their hypofunctions [36].

### 2.2. Older Adults’ Sensory Experiences

The sensory perception capabilities of older adults have great differences from those they had when they were young [37]. Gradual visual degradation and hearing loss are often the main symptoms of the sensory perception deterioration in older adults [38]. In addition, the physiological level of a person’s sensory system sends the stimulus information collected from the outside environment to the brain, and this becomes meaningful experiences of the psychological level [39]. Such sensory experiences are equivalent to the person’s cognition of various events in their daily life, and in this aspect, an older adult in general is not good at conducting complicated work that requires faster body movements, brain reactions, and visual tracking [40,41,42].

One main purpose of this study is to explore the sensory experiences of using the proposed interactive system on slightly disabled elderly people who can move freely with their hands and feet but with relatively slow movements and no jumping, and such a task may be carried out by checking the effects of sensory stimuli and training after the users perform the system.

### 2.3. Design of Man–Machine Interface

“Interaction” means the cognitive feedback of the two sides in a communication process [43]. Lacking experience in using technology products, older adults have more severe problems in manipulating their physiological functions than the young people while using an interaction system [44]. In addition, to encourage elderly people to utilize a technology product in their daily lives, the two main requirements are ease of use and the usefulness of the product [5]. Therefore, to increase older adults’ willingness to use technology products, the best way to design technology products is to satisfy their daily needs and simplify the performance of the products [45,46].

De Schutter and Vanden Abeele [47] investigated the characteristics of games which can attract older adults and which are meaningful to them in life, and induced three core concepts for game designs, namely, connect, cultivate, and contribute, as elaborated in Table 1. Planinc et al. [17] induced eight guidelines for designing exergames that require full-body movement for game control, including: (1) minding the physical condition; (2) using appropriate gestures; (3) avoiding small objects; (4) giving visual and auditive feedbacks; (5) adjusting the difficulty; (6) using a clear user interface; (7) using a suitable topic; and (8) encouraging social interactions. Gerling et al. [48] proposed seven design guidelines for designing exergames for older adults with the aim of promoting their activities and related safety, as shown in Table 2.

Wang and Huang [49] examined usability principles and interface designs in order to understand the relationship between the intentions of mobile e-book interface designs and the users’ perceptions and found that a user’s behavior of operating an interactive interface is related to the user’s prior experience and that the key attributes affecting the user’s behavior of operating an interface include aesthetics, achievement, and friendliness. Schieber [50] studied the interface designs for older adults to overcome the problems they encountered in using advanced technology products that involves the uses of human vision and hearing and proposed design guides such as increasing levels of illumination and minimizing dependence upon peripheral vision; increasing audio stimulus intensity and controlling background noise; avoiding the requirements of detecting acoustic information of high frequencies, etc.

In this study, the principles for interactive system design mentioned in the above review of existing studies, especially those considering the welfare of slightly disabled elderly people, were considered for designing the proposed gaming system, for example, by increasing the brightness and contrast of the game content, enhancing the color richness of the display screen, strengthening the game sound effects to stimulate auditory perception, controlling background music noise, and including proper perception training schemes, etc.

### 2.4. Needs of Gaming Devices for Elderly People’s Amusement and Recreation

With the advance of technology, the development of digital games becomes more and more diversified, increasing their attraction to users of all ages, including children, teens, adults, and even the elderly [51]. The positive effects of digital games brought to older adults include the promotions of their abilities of cognition, hand–eye coordination, concentration, and their feelings of self-confidence [52]. Chandel et al. [53] mentioned that game-based learning is more effective than conventional rote learning. Ijsselsteijn et al. [44] pointed out that an older adult in general has more severe problems than young people in performing physiological functions due to a lack of knowledge and experience of using technology products. Therefore, in the design of a game system for older adults, their vision, hearing, cognition, and body reaction abilities must be taken into consideration seriously.

From the viewpoint of psychological welfare, older adults need amusement and recreation activities for long-term periods at home after they have retired. Specifically, leisure activities can help the elderly to adapt and maintain life satisfaction, improve self-affirmation and emotional relief, enhance physical fitness, and slow down the decline of physical functions. This implies that the older adult needs gerontechnology which is defined by the International Society for Gerontechnology (ISG) as “designing technology and environments for independent living and social participation of older persons in good health, comfort, and safety” [54]. Matsuo et al. [55] found that participation in social activities helps elderly people to have a feeling of healthiness, and Wang [56] indicated that the application of reminiscence therapy is especially appropriate for older people who reside in care facilities and can enrich their lives and promote their health. Hollinworth and Hwang [57] mentioned that in the design of a digital game system, attempts should be made to provide an interactive process similar to real-world events for the older adult to understand the interface operations. Anderson-Hanley et al. [58] found that that for older adults, virtual reality-enhanced interactive exercises by a video exergame (called “cybercycling” that performs stationary cycling with virtual reality tours) two to three times per week for three months yielded greater cognitive benefit and possibly added protection from the progression to mild cognitive impairment when compared with a similar dose of traditional exercise.

In view of all these research findings, the system proposed in this study was designed to provide interactive nostalgic games, which an elderly user can perform to recall his/her past memories after using the system, achieving an effect of reminiscence therapy which is good for the physiological and psychological healthcare of slightly disabled elderly adults.

### 2.5. A Survey of Existing Interactive Systems for Older Adults

It is a trend in recent years that digital media have been used as an effective assistance for promoting the environment for the elderly people to experience interactive technology, achieving the goal of helping them to live a healthier life [17,59,60,61,62,63,64]. In this study several cases of study in this aspect were collected and described in the following.

(1) “Five Touchscreen Tables” [59]

Developed by the Midshire Group of Companies, the five intuitive interactive touchscreen tables can be used to access thousands of downloadable games, puzzles, and quizzes with adjustable difficulties, and they are regarded as perfect tools with sensory applications to provide dementia patients with an engaging familiarity.

(2) “FishCatcher” [17]

Designed according to theoretical guidelines, “FishCatcher” is an exergame for older adults to use with body movements, which is helpful in training of elderly people’s hand–eye coordination.

(3) “Solis” [60]

Implemented by the Build VR group with other companies, “Solis“ is an easy-to-use virtual reality (VR) application created for the aged care industry, which utilizes visual navigation and dual screen accessibility to deliver five video categories relevant to aged care residents, such as wearing a VR helmet to conduct simulated flights to see pretty ground scenes from high above, etc.

(4) “Mobii Interactive Floor/Table” [61]

Designed by the company Omi mainly for people at all stages of dementia, the Mobii Interactive Floor/Table is an in-house, fully portable projection system with one-button height adjustment that can be taken to wherever it is needed and projected onto bedsides, dining tables, and floors, making it an inclusive system with inter-generational appeal.

(5) “Pearls of Life” [62]

Developed by Runk and Pratt Senior Living Communities, the interactive walls of “Pearls of Life” may be used for drawing and gaming, which engages and entertains senior people with Alzheimer’s disease and dementia and creates a multi-sensory experience that keeps the user curious and mentally stimulated.

(6) Rendever’s Virtual Reality Platform [63]

Developed by the Rendever company, the Rendever’s virtual reality platform can transport a user with a headset into an immerse VR experience of taking a stroll down memory lane, for example, by revisiting their childhood homes, wedding locations, or anywhere else from their past.

(7) “NikVision” [64]

Designed by Cerezo et al. [64], “NikVision” is a tangible tabletop that adopts a user-centered approach to the design of therapeutic activities for stimulating older people suffering from cognitive impairments and dementia.

The systems of the cases surveyed above satisfy different application needs of different types of elderly people. For the slightly disabled elderly adults who are the people of main concern in this study, the proposed interactive nostalgic gaming system has the merits of providing simple user interfaces for easy performances by the target audience and possess an uncomplicated structure for fast system development. Such merits promote the happiness and health of elderly people in their aging process.

### 2.6. A Summary of Surveys and Principles Proposed for System Design in This Study

In view of the above surveys of related theories about older adults and existing systems for them, four major principles for gaming system design can be identified, namely, (1) connecting system operations to life experiences; (2) strengthening the perceptual stimulus of the older adults; (3) simplifying operation flows to avoid fast limb and delicate finger manipulations; and (4) appropriately prompting the elderly user on how to operate the system smoothly.

Compared with the systems surveyed previously, the proposed system, which was designed according to the abovementioned four design principles, has the following three additional unique features: (1) use of technology—introducing ICT techniques and the use of digital media into the design of a simple interface for slightly disabled elderly adult; (2) consideration of older adults’ health conditions—designing an interaction process suitable for slightly disabled elderly adults’ capabilities of audiovisual sensing, brain cognition, and hand movement; and (3) inclusion of multiple conceptual factors—considering the factors of nostalgia and entertainment simultaneously in designing the proposed interactive game system.

## 3. Methodology

The details of the proposed methods for prototype system development, the questionnaire survey, and interviews with users and experts conducted in this study, as well as the related ethical considerations, are described in this section.

### 3.1. Prototype Development

Based on the general guidelines for prototype development pointed out in [65,66,67], a prototype of the desired interactive system was constructed and improved to be a final version in this study through the following steps:

(1) Deriving the prototype design principles for use in this study;
(2) Conducting field experiments by inviting users to operate the prototype;
(3) Collecting the users’ opinions about the system usability by interview;
(4) Improving the prototype according to a summary drawn from the opinions; and
(5) Testing the improved version to draw conclusions of this study and suggestions for future studies.

### 3.2. Ethical Considerations

The field experiments of this study were held at the Chang-Tai Elderly Care Center at Huwei Township in Yunlin, Taiwan. The measures taken in the experiments, including the experience of the game process, the interviews, and the questionnaire survey, were approved both by the director of the center, Miss Yi-Shan Kao, and by the participating older adults themselves.

### 3.3. Questionnaire Survey

As mentioned previously, after improving the prototype of the interactive system, a group of older people in the aforementioned elderly care center were invited to play the digital game provided by the system in a second field experiment. A questionnaire survey of the users was conducted to collect their opinions after they finished operating the system. The questionnaire was designed to have simple and concise questions in order to avoid overly guiding the respondents so that the collected answers could be effective [68].

Specifically, the questionnaire includes questions about two aspects, namely, the system usability scale (SUS) [69] and user interaction satisfaction (QUIS) [70]. The questions for both aspects are based on the five-point Likert scale [71], and they will be described in detail later in this paper.

### 3.4. Interviews with Elderly Users

Interviews with the older adults who have played the digital game were conducted in this study. The approach used is the *semi-structured* method [72] which emphasizes that the interviewer has to guide the interviewees dynamically to create open interactions so as to collect diversified comments appropriately. The interview outline designed in this study includes questions asked from three perspectives: users’ operations, users’ feelings, and system design. Because the interviewees are slightly disabled elderly people, the researchers of this study assisted them during the whole interview process. The detailed items of the three aspects used in the interviews will be described later in this paper.

### 3.5. The Interview with Invited Experts

An interview with four invited experts was conducted in this study to observe the system users’ performances and to made comments from four perspectives, namely, user operations, system design, game interface, and future development. Again, the semi-structured method was used in the interview [72]. The experts’ expertise are shown in Table 3, and their comments will be described later in Section 5.

### 3.6. Designs of the Experience Processes in Two Field Experiments

The processes of experiencing the proposed system in the two field experiments conducted in this study were designed to be as follows:

(1) Fields of both experiments—Chang-Tai Elderly Care Center in Taiwan;
(2) Participants—thirty slightly disabled adults with ages over 65 who can use their four limbs freely, but in slower movements.
(3) Times and activities of the first field experiment—30 min with 5 min for listening to the explanation of performing the system, 15 for experiencing the process, and 10 for accepting an interview;
(4) Times and activities of the second field experiment—40 min with 5 min for listening to the explanation of performing the system, 15 for experiencing the interaction process, 10 for accepting a questionnaire survey, and 10 for accepting an interview;
(5) Purposes of the first experiment—understanding the users’ feelings, drawing conclusions from their comments for system improvement;
(6) Purposes of the second experiment—understanding more deeply the users’ feelings from two aspects, system usability and user interaction satisfaction, and drawing conclusions about the effectiveness of the proposed system for future studies.

## 4. System Construction

In this section, the overall system construction will be explained. Specifically, in Section 4.1 the concept of system design is stated, and in Section 4.2 how to design a simple interface is described. The architecture and related components of the proposed system, as well as the detailed nostalgic gaming algorithm are presented in Section 4.3, with an illustration of game play being shown by graphics in Section 4.4. Briefly, the system proposed in this study is an integrated result of cross-field research, combining interactive device design in the engineering field for designing simple interfaces as well as digital media design in the design field, for the purpose of providing nostalgic gaming suitable for slightly disabled elderly adults.

### 4.1. System Design for Nostalgia Gaming

**Figure 1:**
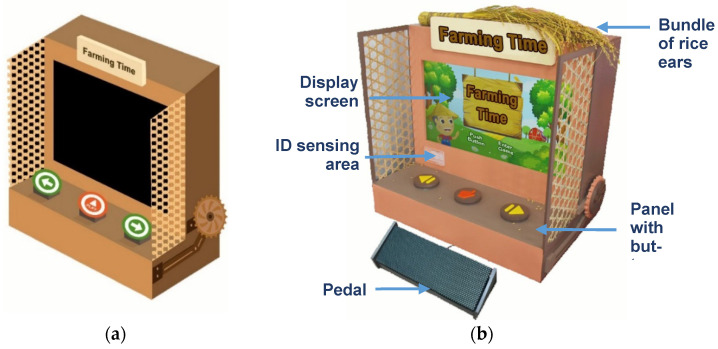
Proposed interactive system simulating an old-time pedal-operated rice threshing machine (**a**) An illustration of the proposed system. (**b**) The actual constructed system.

**Figure 2:**
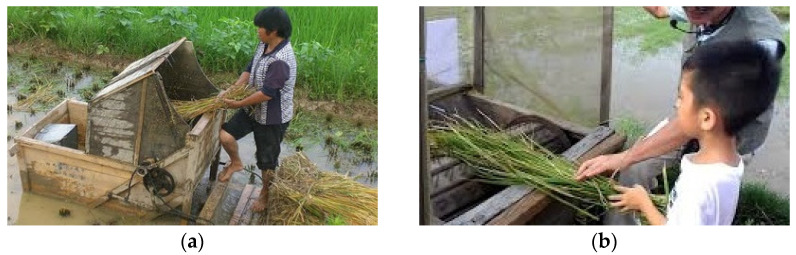
Real old-time pedal-operated rice threshing machines. (**a**) A machine with a complete view [73]. (**b**) Another machine with a closer view [74] (Note: the rice grains are removed away from the rice ears by a roller with nails on the surface, whose rotation is controlled by a pedal stepped on by the farmer).

The proposed interactive system, as shown in Figure 1, was designed to simulate a real old-time pedal-operated rice threshing machine like the one shown in Figure 2a,b [73,74]. Note that by using a real threshing machine, the rice grains can be removed away from the rice ears by a roller with nails on the surface, whose rotation is controlled by a pedal stepped on by a farmer. Three games can be played on the proposed system, each including a random sequence of pedaling and threshing operations simulating the process of rice threshing performed by a farmer on the machine during the rice harvest time.

**Figure 3:**
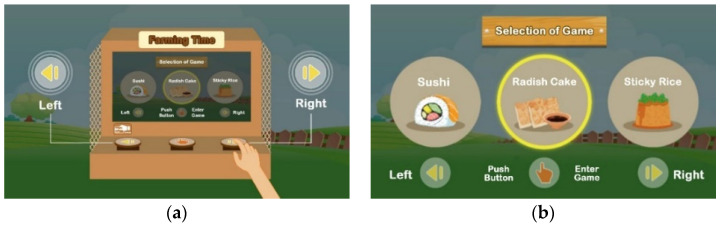
Use of buttons on the tangible panel for selecting and starting games. (**a**) Selecting a game by using the left or right buttons. (**b**) Confirming a selected game and starting it.

**Figure 4:**
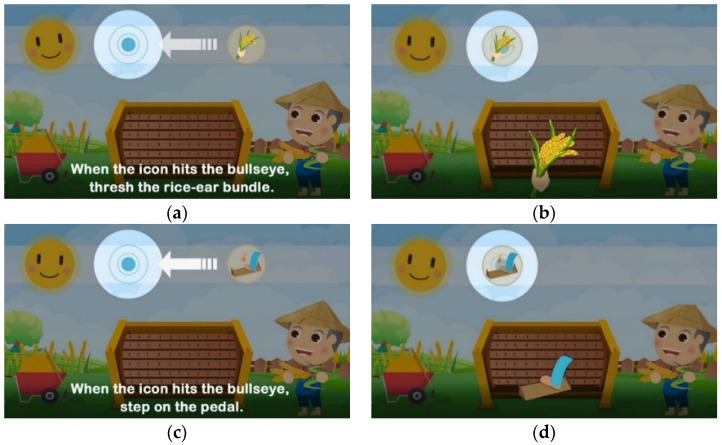
Illustrations on how to play the games to obtain points. (**a**) The icon of “holding the rice ear bundle” is moving to fit into the bullseye. (**b**) While the icon fits well in the bullseye, one point can be obtained by waving the rice ear bundle up and down. (**c**) The icon of “stepping on the pedal” is moving to fit into the bullseye. (**d**) While the icon fits well in the bullseye, one point can be obtained by stepping down on the pedal.

Seen from the exterior, the system as shown in Figure 1b consists of a display screen, a tangible panel with buttons, an ID sensing area, a bundle of rice ears (shown on top of the system), and a pedal (shown below the panel), whose functions are described in the following information:

(1) The display screen—showing the graphics of the intermediate steps of each played game, and the statistics and ranking of the game-playing results (in units of points) of all the users who have ever played games on the machine;
(2) The tangible panel with three buttons—used for playing three games named “sushi,” “radish cake,” and “sticky rice” provided by the system in which, as illustrated by Figure 3, the left and right games may be selected to start by pushing the left and right buttons, respectively, and then the middle one on the panel, while the middle game may be started by pushing the middle button directly without touching the other two buttons;
(3) The ID sensing area—utilized for sensing the ID card of the user playing the game currently and transmitting the ID data to a remote computer to keep;
(4) The bundle of rice ears—held by the user to wave up and down to simulate the rice threshing action at the right time (i.e., at the time when the icon of “holding the rice ear bundle” moves to hit the bullseye) to obtain a point in the game, as illustrated by Figure 4a,b; and
(5) Pedal—stepped on by the user at the right time (i.e., at the time the icon of “stepping on the pedal” moves to hit the bullseye) to obtain a point in the game, as illustrated by Figure 4c,d.

When one of the three games, “sushi,” “radish cake,” and “sticky rice,” is selected and played, the proposed system will generate a corresponding random sequence of two types of icons, namely, “holding the rice ear bundle” and “stepping on the pedal,” for the user to follow to obtain game points according to the ways of threshing and pedaling as described in Figure 4b,d, respectively.

During the above-described scheme of game playing, an elderly user can practice exercises for the mind and body, which include at least the following items:

(1) Conducting visual pattern recognition to identify the two types of icons;
(2) Exerting hand–eye synchronization to move the hand or leg while seeing the icon hit the bullseye; and
(3) Performing hand or leg exercises when threshing or pedaling.

### 4.2. System Architecture

**Figure 5:**
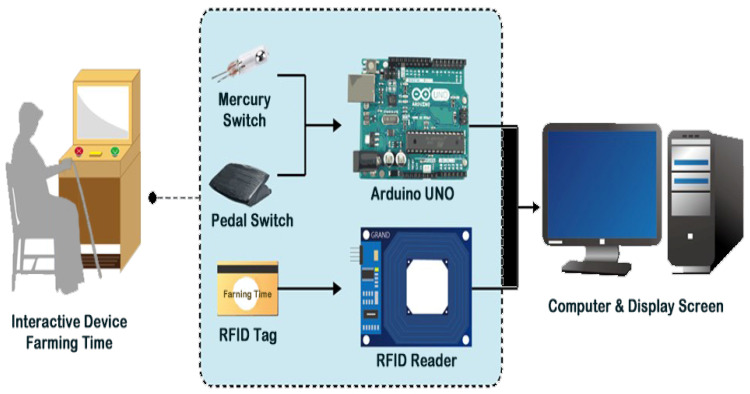
Architecture of the proposed interactive system “Farming Time”.

Figure 5 shows the architecture of the proposed system “Farming Time,” which includes two parts: (1) a software development platform with a display screen built on a computer and programmed using the software Unity3D for game control with the platform functions being presented in the last section; (2) a hardware system including four components: a start button (not shown in Figure 5 but in Figure 3a), a mercury switch, a pedal switch, and a radio frequency identification (RFID) reader with the following functions:

(1) The start button—being in the middle of the panel, this button can be pushed to start the game-playing process;
(2) The mercury switch—being hidden in the bundle of rice ears, this switch can be connected, as the bundle is waved up and down, to send a signal via the Arduino UNO board to the computer to give a game point to the user;
(3) The pedal switch—being hidden in the pedal under the panel of the system, this switch can be connected, while the user steps on the pedal, to send a signal via the Arduino UNO board to the computer to give a game point to the user; and
(4) RFID tag—being included in the user’s ID card, this RFID tag is tapped by the user against the ID sensing area at the start of game play for the content of the tag to be read by the RFID reader to generate the user’s personal data which then are sent to the computer for further processing.

### 4.3. Algorithm of Game Playing

As a summary of the above discussions about playing games on the proposed system “Farming Time,” an algorithm is proposed to describe the details of the game-playing process in the following section, followed by a graphic illustration of an example of running the algorithm as shown in Table 4.

**Table 4:** A graphic illustration of an example of running the game playing process of the algorithm on the proposed system (from the viewpoint of the system using graphic pictures).

Theme No.	Theme of Interaction	Graphics Appearing on the Display Screen	Explanation of Algorithm 1 Steps	Step No.
1	“Initialization” (teaching and inviting of an action)	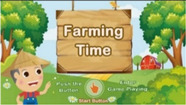	Teach the user to press the start button to start playing the game and wait for the user to proceed as taught until he/she has done so.	1.1 and 1.2
2(a)	“Log in” (teaching)	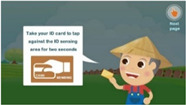	Teach the user to tap the ID card against the ID sensing area for two seconds.	2.1
2(b)	“Log in” (inviting an action)	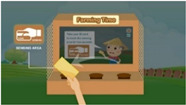	Wait for the user to log in as taught above until he/she has done so.	2.2
2(c)	“Log in” (illustration)	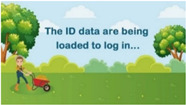	Show the message “The ID data are being loaded to log in…” and save the data in the user database.	2.3
3(a)	“Game selection” (teaching)	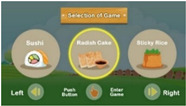	Teach the user to select a game by pressing the left or right button if the middle game is not wanted, and to press the start button to initiate the game.	3.1
3(b)	“Game selection” (inviting an action)	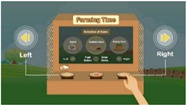	Wait for the user to start a selected game as taught above until he/she has done so.	3.2
3(c)	“Game selection” (illustration)	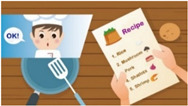	Show the recipe of a dish of food corresponding to the selected game (to lead the elderly user into a mood for game play).	3.3
4(a1)	“Game playing—threshing” (teaching)	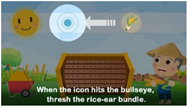	Teach the user to thresh the rice ear bundle to obtain a game point by waving the bundle at the time when the icon of “holding the rice ear bundle” moves to hit the bullseye.	4.1
4(a2)	“Game playing—threshing” (teaching cont’d)	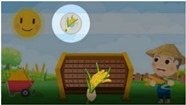	(The same as above.)	4.1 (cont’d.)
4(b1)	“Game playing—pedaling” (teaching)	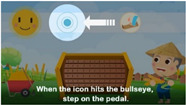	Teach the user to step on the pedal to obtain a game point at the time when the icon of “stepping on the pedal” moves to hit the bullseye.	4.2
4(b2)	“Game playing—pedaling” (teaching cont’d)	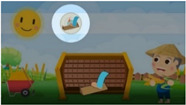	(The same as above.)	4.2 (cont’d.)
4(c)	“Game playing” (inviting actions)	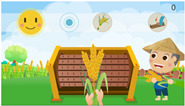	Generate a random sequence of the two icon types and display them one by one and invite the user to play the game by threshing and pedaling as taught above until the sequence is exhausted.	4.3
5(a)	“Game point counting” (illustration)	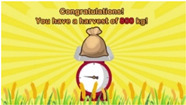	Display the amount of rice grain (in the unit of kg) harvested by the user (each game point = 100 kg).	5.1
5(b)	“Game point counting” (illustration cont’d)	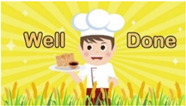	Show the text message “Well done.”	5.2
6	“Ranking of performance” (illustration)	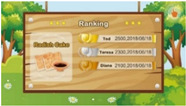	Display the ranking of harvests and related data of all the users having played the selected game in the past according to the weight values of the harvested rice grain.	6

**Algorithm 1** Game play on the system “Farming Time”.**Input:** (1) A user database, with the entry for each user being empty initially; (2) The ID data of the user conducting game play, empty initially; (3) The weight w of the grain threshed by the user, set to zero initially. **Output:** The graphics on the display screen are like those depicted in Table 4. **Steps.** ***//Stage 1: Initialization ―*****Step 1:****//Theme 1: Game initialization** 
1.1Teach the user (by text and audio messages) to press the start button to start the game;1.2Wait for the user to proceed as taught above until he/she has done so;1.3Set grain weight w = 0.
*//Set initial grain weight to be zero***Step 2:****//Theme 2: Log in** 
2.1Teach the user (by text and audio messages) to tap the ID card against the ID sensing area for two seconds;2.2Wait for the user to log in as taught above until he/she has done so;2.3Show the message “The ID data are being loaded to log in…” and save the data in the user database.***//Stage 2: Invitation to select a game ―*****Step 3:****//Theme 3: Game selection**3.1Teach the user (by text and audio messages) to select a game from “sushi,” “radish cake,” and “sticky rice” by pressing the left or right button if the middle game is not wanted, and to press the start button to initiate the game;3.2Wait for the user to start a selected game as taught above until he/she has done so;3.3Show the recipe of a dish of food corresponding to the game selected by the user.*//To lead the user into a mood for game play****//Stage 3: Guidance for game play ―*****Step 4:****//Theme 4: Game play** 
4.1Teach the user (by text and audio messages) to thresh the rice ear bundle to obtain a game point by waving the bundle up and down at the time when the icon of “holding the rice ear bundle” moves to hit the bullseye;4.2Teach the user (by text and audio messages) to step on the pedal to get a game point at the time when the icon of “stepping on the pedal” moves to hit the bullseye;4.3Generate a random sequence of the two types of icons and display them one by one and invite the user (by text and audio messages) to play the game by the actions of threshing and pedaling to obtain game points as taught above until the sequence is exhausted by the user’s actions;4.4For each game point obtained above, set w = w + 50.*//Each game point = 100 kg of grain****//Stage 4: Ending ―*****Step 5:****//Theme 5: Game point counting** 
5.1Display the amount of rice grain (in the unit of kg) harvested by the user (each game point = 100 kg);5.2Show the text message “Well done.”
**Step 6:****//Theme 6: Ranking of performances** Display the ranking of harvests and related data of all the users having played the selected game in the past according to the weight values of the harvested rice grain.*//End*

### 4.4. Illustration of Game Play by Graphics

A table of graphic illustrations of the intermediate graphic outputs of running the above algorithm is given in Table 4, making the stages and steps in the algorithm easier to understand. In the table, the themes mentioned in the algorithm steps are shown in column 2, the graphics yielded by performing the themes and displayed on the screen are shown in column 3, the explanations of the interaction steps are included in column 4, and the step numbers in the algorithm are listed finally in column 5.

## 5. Experiments and Evaluations of Effectiveness of the Proposed System

**Figure 6:**
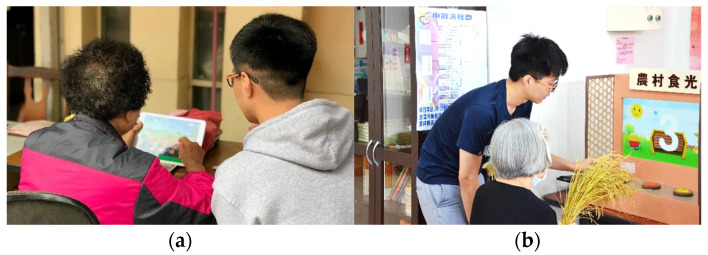
Some pictures of the activities carried out in the two field experiments. (**a**) Helping an older adult to use the prototype system in the first field experiment. (**b**) Explaining the use of the proposed system in the second field experiment.

As mentioned previously, two experiments have been carried out in this study with the first aiming to collect the users’ comments on system performance and modifications and the second aiming to collect opinions from a questionnaire survey, an interview with users, and an interview with four more experts. These opinions are used for evaluating the effectiveness of the modified system. The details are described in the following sections. Two pictures taken during the experiments are shown in Figure 6.

### 5.1. Records of the First Field Experiment for System Modification

In the first experiment, 10 older adults in a daily care center were invited to use the prototype system constructed in this study which was essentially established on an iPad and based on the operation of a waterwheel; their opinions about three aspects of using the prototype, namely, system operation, interactive feeling, and interface design, were collected in the ensuing interviews with them.

According to the collected opinions, the modification of the original prototype includes the following items:

(1) Redesigning the system to be a simulation of an old-time rice threshing machine operated by pedaling;
(2) Creating an installation with a panel, a display screen, and buttons for convenience of game play;
(3) Providing text and audio guidance and graphical illustrations for game play accompanied with music; and
(4) Increasing the fun of game play to allow the selection of a game from three options with a game point ranking scheme. These modifications resulted in the interactive system as described in the last section.

### 5.2. The Records of the Second Field Experiment for System Effectiveness Evaluation

In the second experiment, after the system was used by 30 older adults invited from the previously mentioned daily care center, they were invited to respond to a questionnaire survey for the purpose of evaluating the effectiveness of the proposed system. The questions in the questionnaire included two parts, namely, the SUS and the QUIS, as mentioned previously. That is, the questions were designed from the two aspects of system usability and user interaction satisfaction.

The number of valid questionnaires collected was 27, and the details of the background of the 27 corresponding older adults are listed in Table 5. Specifically, all the participating older adults except one are of ages over 74 and most of them (88.89%) did not have any prior experience of using interactive systems.

### 5.3. Verification of the Reliability and Validity of Collected Questionnaire Survey Data

The *reliability* and *validity* of the collected questionnaire survey data as described above must be verified before they can be used further for evaluating the effectiveness of the proposed system “Farming Time.” For this aim, the statistics software packages SPSS and AMOS were used. The detailed process is described in the Appendix A. As can be seen from the results shown there, it can be concluded that the collected questionnaire survey data for the two aspects of system usability and user interaction satisfaction are both reliable and valid.

### 5.4. Summary of Questionnaire Survey Results

With the collected questionnaire survey data being proven reliable and valid as described above, the contents of the data can be analyzed further to reach the following conclusions.

*(A)*  *Evaluation of the Questionnaire Survey Data on the Aspect of System Usability*

Based on the data shown in Table A5 (Appendix A), the averages of the Likert scores of all the questions of the two scales of ease of use and applicability were computed and are shown in the upper part of Table 6, from which it can be seen that the overall average scores of the two scales are both high up to 4.30 or higher, meaning that the elderly users of the proposed system generally thought the usability and applicability of the system was good. Specifically, regarding the scale of ease of use, the average score was 4.37, indicating that the older adults who have used the proposed system thought that the system was easy to use. In addition, regarding the scale of applicability, the average score was 4.30, meaning that the design of the system was suitable for use by older adults.

An alternative way to evaluate of the effectiveness of the proposed system in the aspect of system usability is to use the Bangor method [75], which computes a score of 0 to 100 to rank the system into five levels, A through F, for the score ranges of 80–100, 70–80, 60–70, 50–60, and 0–49, respectively. The score is computed according to the following steps: (1) subtracting 1 from the score of each positive question; (2) subtracting the score of each negative question from 5; and (3) summing up all the new scores and multiplying the result by 2.5. According to Sauro [76], a score of 68 falling in the range of C or beyond is said to meet the average standard, and a score of 80 falling the range of A is considered as excellent. With the data in Table A5 as the input, the score computed according to the Bangor method for the proposed system is 66.85, which seems to be below the average standard. However, since the number of questions used in this study is not 10 as required by Bangor et al. [75], but rather 8, an additional normalization adjustment by multiplying the computed value by 10/8 was conducted to obtain the final normalized score, which was 83.56, thus falling in the excellent range of A. Therefore, the system is considered very suitable for use by older adults according to Sauro [76].

In more detail, by looking at the questions in Table A5, it can be seen that question A1 was given a relatively low average score of 4.07 (though not lower than 4.0), which means that a few users thought that the interaction scheme of the proposed system was a little bit complicated. This fact might come from the reason that insufficient information about the interface was provided or that the design of the interface is not simple, implying that the operation procedure and associated explanatory information may be improved to be more intuitive for older adults to understand. On the contrary, question A6 was given the high average score of 4.67, which means that most of the users had no trouble in using the interface of the proposed system as long as they understood the operation procedure.

*(B)*  *Evaluation of the Questionnaire Survey Data on the Aspect of User Interaction Satisfaction*

Based on the data shown in Table A7 (Appendix A), the averages of the Likert scores of all the questions of the three scales of goodness to life, ease to learn, and feeling of use were computed and are shown in the lower part of Table 6, from which it can be seen that the overall average scores of all the scales are larger than 4.0, showing that the adult users were generally satisfied with their interactions with the system.

Specifically, as can be seen from Table A7, questions B8 and B9 were given the lowest average scores of 3.85 and 3.74, respectively, which indicates that some users felt it was not so easy to read the information appearing on the display screen and to clearly understand the animation teaching the users about the game. This fact matches with the implication yielded by question A1, mentioned above, about the need to improve the operation procedure for the user to understand the game more easily. One more question given a lower average score of 3.85 is question B13, which implies that the system is not really related to the life experience as expressed by some of the users, and this is reasonable because not all the participating older adults came from rural areas, so some of them did not have the experience of using old-time rice harvesting machines that are operated by the actions of threshing and pedaling as shown in Figure 2.

On the contrary, questions B1, B2, B4, and B11 were given scores higher than 4.60, which shows that most of the elderly users thought that the games built on the system were interesting and easy to play, and they felt relaxed while operating the system and pleasant afterwards.

*(C)*  *Conclusions of the Questionnaire Survey*

As a conclusion, the overall evaluation of the questionnaire survey results using the questions of the two aspects of system usability and user interaction satisfaction listed in Table A5 and Table A7 may be said, as described above, to have proven in general the following results.

(1) The design of the proposed system is good for older adults to use though they have to make a little effort to learn the interaction process of the system initially.
(2) The participating older adults were satisfied with the interaction process as long as they became familiar with the process.
(3) The participating older adults felt relaxed and that it was interesting and pleasant to interact with the proposed system.
(4) The information on introduction to the interface in the form of text and animation may be simplified to be more intuitive for older adults.

### 5.5. Analysis of User Interview Comments for Evaluating the Effectiveness of the Proposed System

After each of the 27 elderly users finished using the proposed system “Farming Time” in the second field experiment, he/she was interviewed by a researcher from this study to collect his/her comments on using the system from three aspects: the operation of the system, feeling of the interaction, and design of the interface. The collected comments are listed in Table 7.

From the interview process, it was found that in general the elderly users had good experiences of interaction with the proposed system and gave positive comments on the design of the interface. However, it is also known that most of the elderly users were afraid to use the interactive system because they were not familiar with the operations of such a kind of system or had not used it before. However, if an elderly user had experience of operating a real rice threshing machine in the past, he/she felt it was handy to use the proposed system. More conclusive remarks can be drawn from the records of the users’ comments listed in Table 7 as described in the following section:

(1) The slightly disabled elderly users felt a little bit of pressure when they started to operate the system, but they became more relaxed after continuing to interact with the system.
(2) Animated prompts are more helpful to the user’s operation than text prompts.
(3) The slightly disabled elderly users were less proficient in facing choices when operating the interactive system.
(4) It was more difficult for the elderly to control the system if more than two operations were required at a time, such as using hands and feet in the meantime.
(5) It is easier for the slightly disabled elderly users with farming experiences to understand the design concept of the system.

### 5.6. Analysis of Interview Comments from the Experts

In the second field experiment, as mentioned previously, four experts were additionally invited to observe the slightly disabled elderly users playing the game. At the end of this experiment, the experts were interviewed to collect their comments about four aspects: user operations, system design, game interface, and future development. The comments collected are summarized in the following:

(1) It is a suitable way to encourage elderly people’s willingness to perform exercises using an interactive system;
(2) Elderly people are more willing to accept activities and objects which are related to their experiences;
(3) Elderly people can train their cognition and motor capabilities by using an interactive system;
(4) Whether the system can be operated smoothly can be adopted as a pre-test for the slightly disabled elderly people to find out whether their physical and mental functions are degraded; and
(5) The interfaces of the system should be easy to use, requiring no complicated limb functions of the elderly individuals with mild disabilities.

## 6. Discussion and Conclusions

### 6.1. Discussion

The participants of the experiments conducted in this study are slightly disabled elderly adults with ages over 65 at Chang-Tai Elderly Care Center in Taiwan. From the qualitative and quantitative statistical analyses of the data collected from the questionnaire survey and interviews carried out after the experiments were completed, as described previously, the following facts can be noted.

(1) The digital game played by the slightly disabled elderly people creates positive influences on the effect of active aging.

According to the questionnaire survey and interview results, most slightly disabled elderly users gave positive evaluations of the proposed interactive system, indicating that they felt relaxed and interested when using the system and their limb mobility and cognitive abilities were improved, with all these factors being helpful to active aging.

(2) The participants become more willing to play digital games after performing the proposed system.

The games offered by the proposed interactive system “Farming Time” were designed for use by slightly disabled older adults with the functions of nostalgia and entertainment. During the interactions, the elderly users were seen to be interested in using the system, and through observations and interviews, it is understood that the slightly disabled elderly users were more willing to try the interactive system and felt satisfied.

(3) Enhancement of the perceptual experience of playing the digital game by dynamic audio and visual effects can attract the attention of the slightly disabled users during the interactive process.

When operating the system, the slightly disabled older adult might have difficulty in performing the interaction steps because of unfamiliarity with the interaction process or unclear information about the interface scheme. It was found in this study through user interviews that elderly users will increase their attention during the interaction process if more dynamic visual or sound prompts are added into the interface scheme.

(4) Slightly disabled older adults have higher degrees of acceptance of technological products if the products are related to their own living experiences.

Because elderly people are less familiar with the operation of interactive systems, they feel worried and scared when using them for the first time. If the system can be designed to be based on objects that are familiar to them, such as old-time machines, then, regardless of the operation method and interface style, older adults tend to accept the system more easily with fewer worries.

(5) Playing the digital game has the effect of training the slightly disabled elderly users’ cognitive and motor abilities, achieving the effects of stimulating the senses and strengthening the body and mind.

Through observations and interviews, it was found that during the interaction with the system, slightly disabled older adults can stimulate their visual senses by watching the game interface prompts, and move the muscles of the hands and feet in the meantime to achieve hand–eye coordination when playing the digital game.

### 6.2. Conclusions

A simple interactive system for nostalgic gaming to promote the health of slightly disabled elderly people has been proposed in this study. The system is constructed to simulate a rice threshing machine used by people in earlier times. The system was designed according to the principles derived from literature reviews and refined according to the comments of invited older users who used the system. The refined system was then performed by a group of invited slightly disabled elderly people. A questionnaire survey was conducted to collect their opinions after their interactions with the system. Finally, the opinion data were analyzed by the statistical packages of SPSS and AMOS with the concluding remarks of being reliable and valid.

Interviews with the aforementioned system’s users and several invited experts were also conducted to collect their comments on system usability, which were then evaluated to reveal several findings about the system’s effectiveness as described in the following section:

(1) Slightly disabled elderly people are more willing to accept digital interactive systems for gaming involving their life experiences, which are helpful for them to achieve active aging.
(2) The system interfaces should be simple and tangible, requiring no complicated limb functions of the elderly adults with mild disabilities.
(3) Digital game play is effective for training the cognitive and motor abilities of the slightly disabled elderly people and strengthening their body and mind.

In the future, more types of interactive systems for improving other aspects of elderly people’s welfare may be developed and evaluated. In addition, the sample group involved in the experiments of this study is small in size because only 30 slightly disabled older adults were being taken care of in the Chang-Tai Elderly Care Center where the experiments were conducted; attempts will be made to find a larger sample group for the experiments in future studies. Attempts may also be made to adopt more advanced digital interactive techniques for the elderly people, helping create friendlier LOHAS environments for active aging.

## Figures and Tables

**Table 1:** Principles for designing meaningful games proposed by De Schutter and Vanden Abeele [47].

Concept	Principle
Connect	Emphasizing connectedness to the significant other, children, grandchildren and friends, and society, e.g., via multiplayer options, extra forums, themes about family members, etc.
Cultivate	Cultivating oneself or others to gain knowledge and allowing seniors to share their wisdom with other players, e.g., by adding content to a game, quizzing, etc.
Contribute	Trying to contribute to society in a larger context and helping people ahead with the aim of creating a better world.

**Table 2:** Principles for designing exergames for the older adult proposed by Gerling et al. [48].

Concept	Principle
Age-inclusive design	Creating inclusive games by embracing age-related physical and cognitive impairments
ROM-adaptability	Creating interaction paradigms that adapt to individual differences in player range of motion
Exertion management	Providing fatigue management and preventing overexertion by appropriate game pacing
Dynamic game difficulty	Offering difficulty adjustments between players and individually scaling challenges
Easy gesture recall	Providing natural mappings and clear instructions that support gesture recall to empower players
Continuous player support	Integrating continuous tutorials and player promotion to facilitate gesture learning and interaction
Simple setup routines	Implementing easy menus, startup, and shutdown routines to encourage independent play

**Table 3:** List of the backgrounds of the invited experts interviewed in this study.

No.	Organization	Occupation	Expertise
A	Care center for older adults	Social worker	Design of activities and care service for older adults
B	LOHAS service center for the elderly	Center director	Psychology and care service of aging adults
C	Local hospital	Physician	General medical disease, older adults’ disease
D	National university	Professor	User interface design, cognitive psychology, aging-related research

**Table 5:** Statistics of the backgrounds of the elderly users in the second field experiment.

Category	Item	Number of Samples	Percentage (%)
Gender	Male	7	25.93
	Female	20	74.07
Age	65~74	1	3.70
	75~84	17	62.96
	85~	9	33.34
Exercise habit	Often	13	48.14
	Sometimes	7	25.93
	Almost never	7	25.93
Experience of playing interactive games	Often	0	0
	Sometimes	3	11.11
	Almost never	24	88.89

**Table 6:** Statistics of the average Likert scores of the scales of the aspect of system usability and user interaction satisfaction.

Aspect	Scale	Average
System usability	Ease of use	4.37
	Applicability	4.30
User interaction satisfaction	Goodness to life	4.18
	Ease to learn	4.17
	Feeling of use	4.50

**Table 7:** The list of comments on the proposed system collected from interviews with the slightly disabled elderly users in the second field experiment.

Aspect	Question	Record of Interview Comments
Operation of the system	Do you know how to play this system?	(1) The user knew how to play, just like the action of threshing rice conducted before. (2) The user was not clear about how to play the game at first and felt scared before the operation, but after a few practice sessions, he/she could operate it by himself/herself.
	Is there anything that you do not understand or that you think needs improvement when you operate this interactive system? For example?	(1) The user indicated that the foot pedaling part cannot be followed immediately in the operation process. (2) The user said that the teaching animation is not clear enough. (3) The user said that the left and right buttons are difficult to understand, and the gameplay level is difficult to choose.
	Do you like the design of the interface of the interactive system? Why?	(1) The user likes the interface design of the system. (2) The user thought that the interactive style is novel and interesting. (3) The user said that the color of the game screen looks comfortable.
Feeling of the interaction	Do you feel happy during the interactive experience process? Why?	(1) The user felt very happy during the interactive experience process with his/her spirits being lifted. (2) The user hoped that the gameplay of the rice threshing machine could be more exciting so that the experience can be more like using a real machine.
	Will you want to play on the system again? Why?	(1) The user would continue to play on the interactive system and would prefer it to general sports equipment. (2) This interactive system is less laborious and can be played for a longer time. (3) The user would like to play again and would share the interactive experience of using the system with his family
	What do you think or feel about the system after using it?	(1) The sound prompt is mixed with the background music, which is not easy to hear clearly. (2) The ears of rice are too light to hold with a feel of weight.
Design of the interface	Do you think of rural memories during the interactive experience process or of other things?	(1) The system is very much like the old-time rice threshing machine (2) The pedal of the threshing machine is like that of a sewing machine.

**Figure A1:**
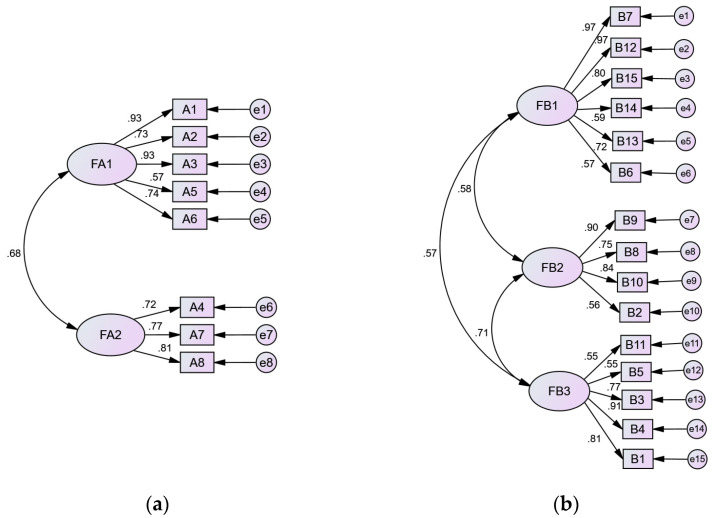
The graphs of the data structure models yielded by the CFA of the two aspects of system usability and user interaction satisfaction. (**a**) The graph of the structure model of the aspect of system usability (FA1: ease to learn; FA2: applicability). (**b**) The graph of the structure model of the aspect of user interaction satisfaction (FB1: goodness to life; FB2: ease to learn; FB3: feeling of use).

## Data Availability

The datasets used and/or analyzed during the current study are available from the corresponding author on reasonable request.

## Appendix A. Verification of the Reliability and Validity of Collected Questionnaire Survey Data

The following process is conducted in this study using the statistics software packages SPSS and AMOS to verify the reliability and validity of collected questionnaire survey data.

(1) Testing the adequacy of the collected data—using the SPSS (Statistical Product and Service Solutions) to conduct the Kaiser–Meyer–Olkin (KMO) test and Bartlett’s test of sphericity.
(2) Finding the latent scales of the questions created for collecting data in the questionnaire survey—using the SPSS to perform exploratory factor analysis (EFA) via principal component analysis (PCA) as well as the varimax rotation method with Kaiser normalization.
(3) Verifying the reliability of the data collected from the questionnaire survey—using the values of Cronbach’s **α** coefficients values computed by the EFA process.
(4) Verifying the suitability of the model of the data setup structure according to the latent scales found in Step 2 above—using the software package AMOS to perform confirmatory factor analysis (CFA).
(5) Verifying the validity of the collected questionnaire survey data—based on a number of parameter values created by the EFA and CFA using the SPSS and AMOS.

The above five steps for verifying the reliability and validity of the collected questionnaire survey data are described in detail in the following.

(A) *Testing the Adequacy of the Collected Questionnaire Survey Data*

To evaluate the adequacy of the collected questionnaire survey data, the Kaiser–Meyer–Olkin (KMO) test and the Bartlett’s test of sphericity were adopted in this study [77,78,79,80,81,82]. The KMO measure is a statistic used to indicate the proportion of the variance among the variables that might be caused by certain factors underlying the variables. A KMO measure value larger than the threshold value 0.50 is usually taken to be acceptable to pass the test [79,80]. In addition, the Bartlett’s test of sphericity is employed to decide whether the data variables are uncorrelated or not. A significance value yielded by the test smaller than the threshold of 0.05 is in general acceptable to decide that the data variables are correlated, and the test is said to be passed [81]. When both tests are passed, the data variables are regarded to be related sufficiently for a further step of structure analysis [82].

With the collected questionnaire survey data listed in Table A1 and Table A2 as inputs, the values of the KMO measure and the significance of the Bartlett’s test for the two aspects of system usability and user interaction satisfaction yielded by the SPSS are shown in Table A3. It can be seen from the table that, for either of the two aspects, the KMO measure is larger than the threshold value 0.5 and the significance of the Bartlett’s test is smaller than the threshold value 0.05. Consequently, it may be decided that the data of each of the two aspects are adequately related for further data structure analysis, as described next.

**Table A1:** Statistics of the Likert scores of the users’ answers to the questionnaire survey for the aspect of system usability (unit: %; score: 1~5).

Question	StronglyDisagree	Disagree	No Opinion	Agree	Strongly Agree
	(1)	(2)	(3)	(4)	(5)
A1. I think the interaction scheme of “Farming Time“ is too complicated. *	0.00	7.41	11.11	48.15	33.33
A2. I think “Farming Time” is easy to operate.	0.00	0.00	3.70	48.15	48.15
A3. I need repeated explanations of the operation steps before I can use the interactive interface. *	0.00	7.41	3.70	48.15	40.74
A4. I think “Farming Time” has many inconsistencies in its interactive interface. *	0.0	0.0	14.82	40.74	44.44
A5. I can learn to use the interface of “Farming Time” as quickly as most of the other users.	0.0	3.70	0.0	44.45	51.85
A6. I think it troublesome to use the interface of “Farming Time.” *	0.00	0.00	7.41	18.52	74.07
A7. I am confident that I can operate “Farming Time.”	0.00	0.00	11.11	59.26	29.63
A8. I have to learn a lot of extra information before I can use “Farming Time.” *	0.00	0.00	3.70	51.85	44.45

* The asterisked questions in the table are negative in meaning and their scores have been inverted to match those of the positive questions.

**Table A2:** The five-scale Likert scores of the users’ answers of the questionnaire survey for the aspect of user interaction satisfaction (unit: %; score: 1~5).

Question	Strongly Disagree	Disagree	No Opinion	Agree	Strongly Agree
	(1)	(2)	(3)	(4)	(5)
B1. The interactive process is interesting.	0	0	7.41	22.22	70.37
B2. The system play process is fluent.	0	0	0	25.93	74.07
B3. The system is attractive.	0	3.7	11.11	40.74	44.45
B4. I feel pleasant after using the system.	0	3.7	3.7	29.63	62.97
B5. The game screen is visually abundant.	0	0	3.7	51.85	44.45
B6. The audio effect of the game is rich.	0	0	3.7	77.78	18.52
B7. The system is helpful to coordinate vision with hands and legs.	0	7.41	0	40.74	51.85
B8. It is easy to read the information appearing on the display screen.	0	7.41	18.52	55.55	18.52
B9. It is easy to understand the teaching animations in the game play process.	0	14.81	14.81	51.85	18.53
B10. It is easy to understand the audio prompts in the game play process.	0	3.7	3.7	48.15	44.45
B11. I feel relaxed while operating the system.	0	0	3.7	25.93	70.37
B12. The system is helpful to my daily life.	0	7.41	0	40.74	51.85
B13. The system is related to my life experience.	3.7	0	25.93	48.15	22.22
B14. After experiencing the system, I feel more active to get along with other people.	0	0	14.81	59.26	25.93
B15. After experiencing the system, I feel more willing to participate in social activities.	3.7	0	7.41	48.15	40.74

**Table A3:** The KMO measure values and the significance values of the Bartlett’s test of the collected questionnaire survey data of the two aspects of system usability and user interaction satisfaction.

Aspect	Measure/Test		Value
System usability	Sampling adequacy measures yielded by the KMO test		0.817
	Bartlett’s test of sphericity	Approximate Chi-Square value	119.278
		Degree of freedom	28
		Significance	0.000
User interaction satisfaction	Sampling adequacy measures yielded by the KMO test		0.752
	Bartlett’s test of sphericity	Approximate Chi-Square value	264.193
		Degree of freedom	105
		Significance	0.000

(B) *Finding the Latent Scales of the Questions Used in Questionnaire Survey Data*

With the adequacy of the questionnaire survey data being verified as above, by taking the data in Table A1 and Table A2 as inputs, the SPSS were employed to perform EFA by PCA and the varimax rotation method with Kaiser normalization to find possible groups of related questions for each aspect (i.e., for the aspect of either system usability or user interaction satisfaction), with each group possessing a common property called scale.

Specifically, for the aspect of system usability, after the EFA process was conducted, the rotated matrix shown in Table A4 is yielded. From the table, it can be seen that the questions are categorized into two groups FA1 = (A1, A3, A6, A2, A5) and FA2 = (A8, A7, A4) according to the clustering results of the factor loading values in the matrix. The two groups, regarded to belong to two scales, are given the titles of ease of use and applicability, respectively, in this study. Including these two scales into Table A1 and replacing the Likert score values in the table by their statistics (minimum, maximum, average, and standard deviation) results in a new table, namely, Table A5 which will be utilized later for the evaluation of the effectiveness of the proposed gaming system.

**Table A4:** The rotated component matrix yielded by EFA to generate two scales of questions of the aspect of system usability.

Rotated Component Matrix ^a^		
Question	Component (scale)	
	1	2
A1	0.877	0.334
A3	0.874	0.335
A6	0.839	0.093
A2	0.618	0.529
A5	0.579	0.241
A8	0.139	0.892
A7	0.301	0.791
A4	0.307	0.728

^a^ Extraction method: principal component analysis; rotation method: varimax with Kaiser normalization; convergence: rotation converged in three iterations.

**Table A5:** Statistics of the Likert scores of the users’ answers of the questionnaire survey for the aspect of system usability (S.D.: standard deviation).

Scale	Question	Min.	Max.	Average	S. D.
Ease of use	A1. I think the interaction scheme of “Farming Time“ is too complicated. *	2	5	4.07	0.87
	A3. I need repeated explanations of the operation steps before I can use the interactive interface. *	2	5	4.22	0.85
	A6. I think it troublesome to use the interface of “Farming Time.” *	3	5	4.67	0.62
	A2. I think “Farming Time” is easy to operate.	3	5	4.44	0.58
	A5. I can learn to use the interface of “Farming Time” as quickly as most of the other users.	2	5	4.44	0.70
Average				4.37	
Applicability	A8. I have to learn extra information before I can use “Farming Time.” *	3	5	4.41	0.57
	A7. I am confident that I can operate “Farming Time.”	3	5	4.19	0.62
	A4. I think “Farming Time” has many inconsistencies in its interactive interface. *	3	5	4.30	0.72
Average				4.30	

* Note: the asterisked questions in the above table are negative and their scores have been inverted to match those of the positive questions.

Similarly, for the aspect of user interaction satisfaction, the EFA yields Table A6 from which the questions can be seen to be categorized into three groups FB1 = (B7, B12, B15, B14, B13, B6), FB2 = (B9, B8, B10, B2), and FB3 = (B11, B5, B3, B4, B1) according to the clustering results of the factor loading values in the matrix. The three groups, regarded to belong to three scales, are given the titles of goodness to life, ease to learn, and feeling of use, respectively, in this study. Including these three scales into Table A2 and replacing the Likert score values in the table by their statistics results in a new table, namely, Table A7, which will also be used later for evaluating the effectiveness of the proposed system.

**Table A6:** The rotated component matrix yielded by EFA to generate three scales of questions of the aspect of user interaction satisfaction.

Rotated Component Matrix ^a^			
Question	Component (scale)		
	1	2	3
B7	0.877	0.286	0.117
B12	0.875	0.329	0.084
B15	0.785	0.223	0.393
B14	0.689	0.146	0.100
B13	0.676	0.269	0.283
B6	0.665	−0.057	0.190
B9	0.277	0.841	0.180
B8	0.041	0.831	0.150
B10	0.205	0.745	0.401
B2	0.416	0.574	0.076
B11	0.227	−0.074	0.781
B5	−0.015	0.255	0.699
B3	0.322	0.332	0.689
B4	0.324	0.417	0.681
B1	0.348	0.492	0.548

^a^ Extraction method: principal component analysis; rotation method: varimax with Kaiser normalization; convergence: rotation converged in three iterations.

**Table A7:** Statistics of the average Likert scores of the users’ answers of the questionnaire survey for the aspect of user interaction satisfaction (S.D.: standard deviation).

Scale	Question	Min.	Max.	Average	S. D.
Goodness to life	B7. The system is helpful to coordinate vision with hands and legs.	2	5	4.37	0.84
	B12. The system is helpful to my daily life.	2	5	4.37	0.84
	B15. After experiencing the system, I feel more willing to participate in social activities.	1	5	4.22	0.89
	B14. After experiencing the system, I feel more active to get along with other people.	3	5	4.11	0.64
	B13. The system is related to my life experience.	1	5	3.85	0.91
	B6. The audio effect of the game is rich.	3	5	4.15	0.46
Average				4.18	
Ease to learn	B9. It is easy to understand the teaching animations in the game play process.	2	5	3.74	0.94
	B8. It is easy to read the information shown on the display screen.	2	5	3.85	0.82
	B10. It is easy to understand the audio prompts in the game play process.	2	5	4.33	0.73
	B2. The system play process is fluent.	4	5	4.74	0.45
Average				4.17	
Feeling of use	B11. I feel relaxed while operating the system.	3	5	4.67	0.56
	B5. The game screen is visually abundant.	3	5	4.41	0.57
	B3. The system is attractive.	2	5	4.26	0.81
	B4. I feel pleasant after using the system.	2	5	4.52	0.75
	B1. The interactive process is interesting.	3	5	4.63	0.63
Average				4.50	

(C) *Verifying the Reliability of the Questionnaire Survey Data by the Cronbach’s* *α Coefficients*

Reliability is defined as a measure of the consistency of a data set despite the repeated times [83]. The Cronbach’s α coefficient [84,85] computed by the aforementioned EFA was employed in this study to analyze the reliability of the collected questionnaire survey data. The closer the Cronbach’s α coefficient of a data set of a scale to the value of 1.0, the greater the reliability of the data set. Based on Gilford [86], the goodness of the reliability of a data set can be decided according to the following rules:

*α* ≥ 0.70 → highly reliable;

0.35 ≤ *α* < 0.70 → reliable;

*α* ≤ 0.35 → unreliable

where *α* is the Cronbach’s α coefficient value.

The Cronbach’s α coefficient values yielded by the EFA of the questionnaire survey questions of the two scales, ease of use and applicability, of the aspect of system usability as well as those of the questionnaire survey questions of the three scales, goodness to life, ease to learn, and feeling of use, of the aspect of user interaction satisfaction are all listed in Table A8. The coefficient values of the five scales and those of the two aspects can all be seen to lie in the range of 0.35 to 0.70 or even larger as shown in the table, indicating that the collected questionnaire survey data are reliable.

**Table A8:** The Cronbach’s α coefficients of the collected questionnaire survey data of the scales and aspects of the questions.

Aspect	Scale	Cronbach’s α Coefficient of the Scale	Cronbach’s α Coefficient of the Aspect
System usability	Ease of use	0.879	0.890
	Applicability	0.796	
User interaction satisfaction	Goodness to life	0.901	0.920
	Ease to learn	0.835	
	Feeling of use	0.844	

(D) *Verifying the Suitability of the Model Structure of the Collected Data Set Up by the Found Scales*

To prove the validity of the questionnaire survey data, the suitability of the structure models of the data of the two aspects set up by the found scales must be verified [87]. For this purpose, the confirmatory factor analysis (CFA) process using the software package AMOS was applied on the collected data, yielding two structure-model graphs, one graph with two scales and the other with three, as shown in Figure A1 in which the values besides the arrows are standardized regression weights. The graphs will be used later for proving the validity of the collected questionnaire survey data.

In addition, a list of structure model fit indices was yielded as well by the CFA for each aspect, including the degrees of freedom (df), the chi-square (χ^2^) statistics, the ratio χ^2^/df, the comparative fit index (cfi), the adjusted goodness-of-fit index (agfi), and the root mean square error of approximation (RMSEA), as shown integrally in Table A9. From the table, the following observations about the goodness of fit of the model structure set up by the found latent scales to the collected questionnaire survey data can be made:

(1) The small values, 0.96 and 1.05, of χ^2^/df (the ratio the value of χ^2^ to the degree of freedom df) mean that the model structures set up by the scales of the two aspects are both of a good fit to the collected data because a value of χ^2^/df smaller than 3 and larger than 1 means that the model has a good-fit structure according to Hair et al. [88];
(2) The cfi values, 1.00 and 0.98, also show that the models are good in structure because a cfi value around 0.90 or larger means that the model structure has a reasonable goodness of fit according to Bentler [89];
(3) The agfi values, 0.76 and 0.62, are smaller than the threshold 0.9 usually taken to judge a good-fit model structure; however, as mentioned in MacCallum and Hong (1997), when the number of samples is not large like the case of this study here, the computed agfi values of a good-fit model structure can become around or even smaller than 0.8 according to MacCallum and Hong [90]; and
(4) The RMSEA values, 0.00 and 0.05, mean that the model structures are reasonably good according to Hu and Bentler [91] in which it is pointed out that an RMSEA value no larger than 0.06 indicates that a reasonable fit of the model structure to the collected data.

All the above facts indicate that the model structures set up by the found scales of the two aspects of system usability and user interaction satisfaction have a reasonable fit to the collected questionnaire survey data.

**Table A9:** The fit indices of the data structure models of the two indicators of system usability and user interaction satisfaction computed by CFA ^a^.

Scale	df	χ^2^	χ^2^/df	cfi	agfi	RMSEA	RMSEA (90% CI)	
							Low	High
System usability	19	18.18	0.96	1.00	0.76	0.00	0.00	0.16
User interaction satisfaction	87	91.62	1.05	0.98	0.62	0.05	0.00	0.12

^a^ df: the degree of freedom; cfi: comparative fit index; agfi: average gfi; RMSEA: root mean square error of approximation; CI: confidence interval.

(E) *Proving the Validity of the Questionnaire Survey Data by EFA and CFA*

With the model structures of the two aspects of questionnaire questions both being proven to fit reasonably to the collected questionnaire survey data, it is appropriate to further analyze the validity of the data. The structure models in Figure A1 show that the standardized regression weights with respect to the scales (appearing on the paths from scales FA1 and FA2 to questions A1–A8 and from scales FB1–FB3 to questions B1–B15) are all larger than the threshold of 0.5, indicating that the construct validity of the model was verified [92,93]. This fact may be shown as well by the construct validity values of all the scales of the two aspects of system usability and user interaction satisfaction computed by the aforementioned EFA process, which are listed in Table A10 and can be seen to be all larger than the threshold of 0.6 [92,93]. This completes the proof of the construct validity of the collected questionnaire survey data of the indicator of system usability.

**Table A10:** The values of construct validity of the scales of the two aspects of system usability and user interaction satisfaction computed by the CFA.

Aspect	Scale	Group of Related Questions	Construct Validity Value
System usability	Ease of use	(A1, A3, A6, A2, A5)	0.891
	Applicability	(A8, A7, A4)	0.811
User interaction satisfaction	Goodness to life	(B7, B12, B15, B14, B13, B6)	0.903
	Ease to learn	(B9, B8, B10, B2)	0.853
	Feeling of use	(B11, B5, B3, B4, B1)	0.848
